# Involvement of ESCRT-II in Hepatitis B Virus Morphogenesis

**DOI:** 10.1371/journal.pone.0091279

**Published:** 2014-03-10

**Authors:** Jens T. Stieler, Reinhild Prange

**Affiliations:** Department of Medical Microbiology and Hygiene, University Medical Center of the Johannes Gutenberg University Mainz, Mainz, Germany; Yonsei University, Republic of Korea

## Abstract

The hepatitis B virus (HBV) is an enveloped DNA virus that replicates via reverse transcription of its pregenomic RNA (pgRNA). Budding of HBV is supposed to occur at intracellular membranes and requires scission functions of the endosomal sorting complex required for transport (ESCRT) provided by ESCRT-III and VPS4. Here, we have investigated the impact of the upstream-acting ESCRT-I and ESCRT-II complexes in HBV morphogenesis. RNA interference knockdown of the ESCRT-I subunits TSG101 and VPS28 did not block, but rather stimulate virus release. In contrast, RNAi-mediated depletion of the ESCRT-II components EAP20, EAP30 and EAP45 greatly reduced virus egress. By analyzing different steps of the HBV maturation pathway, we find that the knockdown of ESCRT-II not only inhibited the production and/or release of enveloped virions, but also impaired intracellular nucleocapsid formation. Transcription/translation studies revealed that the depletion of ESCRT-II neither affected the synthesis and nuclear export of HBV-specific RNAs nor the expression of the viral core and envelope proteins. Moreover, the absence of ESCRT-II had no effects on the assembly capability and integrity of HBV core/capsids. However, the level of encapsidated pgRNA was significantly reduced in ESCRT-II-depleted cells, implicating that ESCRT-II directs steps accompanying the formation of replication-competent nucleocapsids, like e.g. assisting in RNA trafficking and encapsidation. In support of this, the capsid protein was found to interact and colocalize with ESCRT-II subunits in virus-producing cells. Together, these results indicate an essential role for ESCRT-II in the HBV life cycle and suggest that ESCRT-II functions prior to the final HBV budding reaction.

## Introduction

An essential step in the formation of an extracellular enveloped virus particle is the separation of virus and host cell membranes. For many viruses, this requires the recruitment of a network of proteins normally involved in two analogous cellular membrane fission events, the budding of cargo-containing vesicles into multivesicular bodies (MVBs) and the separation of daughter cells during cytokinesis. This network, collectively called ESCRT (endosomal sorting complex required for transport), consists of heteromeric ESCRT-0, -I, -II, and -III complexes together with the VPS4 ATPase that seem to function in a sequential manner [1]–[4]. In the course of MVB biogenesis, the ESCRT machinery is essential for the sorting of cargo proteins into intraluminal vesicles either for degradation, lysosomal functions, or exosomal release [2], [5]. Generally, monoubiquitination serves as a signal for ESCRT-mediated cargo sorting [2], [4]. The different ESCRT complexes perform specific functions in this process: the early-acting factors ESCRT-0 and ESCRT-I comprise ubiquitin-binding components and recognize and concentrate cargoes, whereas the later-acting factor ESCRT-III mediate membrane constriction and scission events and recruit the terminal VPS4 ATPase complex for disassembly and recycling of the ESCRT machinery [1], [3], [5], [6]. ESCRT-II is supposed to connect the upstream cargo-binding components of the system with the downstream membrane remodeling system [7]–[9]. However, unlike yeast ESCRT-II, the role of mammalian ESCRT-II in MVB sorting remains to be unambiguously established, as there are contradictorily reports for its requirement in the downregulation of MVB cargoes [8], [10], [11]. During cytokinesis, only subsets of ESCRT act with ESCRT-II being presumably dispensable [12], [13]. Similarly, enveloped viruses recruit some of the ESCRT machinery through the function of specific peptide motifs within their structural proteins referred to as late assembly domains [14]–[18].

All viruses that bud in an ESCRT-dependent manner share the requirement for the membrane abscission function provided by ESCRT-III and VPS4, but differ in their need for upstream-acting factors. Viruses relying on P(S/T)AP late motifs engage the pathway via interaction with the ESCRT-I component TSG101 [15], [16], [19], [20]. The (L)YPX_n_L-type late domains bind and recruit Alix, an ESCRT-associated protein that links viral proteins to the CHMP4 subset of the ESCRT-III complex [15], [16], [21]. Connections between PPXY-type late domains and the ESCRT machinery are less firmly established, although these motifs constitute ligands for Nedd4-like HECT ubiquitin ligases that are normally required for tagging membrane proteins for MVB sorting and lysosomal degradation [4], [5], [15]–[18]. Irrespective of the particular late domain and the accordingly entry site into the ESCRT pathway, the ESCRT-II complex seems to be largely dispensable for enveloped virus budding [8], [22]–[24].

The hepatitis B virus (HBV) is an enveloped, DNA-containing pararetrovirus that requires ESCRT-III and VPS4 to exit cells [25]–[27]. Upon infection of liver cells, the partially double-stranded 3.2-kb DNA genome is converted to the covalently closed circular DNA inside the nucleus. In cell culture, this process can be mimicked by transfection with replication-competent HBV genomes. The genome serves as a template for the transcription of the pregenomic (pg) RNA and three subgenomic RNAs that are exported to the cytoplasm. The pgRNA is bifunctional, as it represents the message for the core protein and the polymerase (P), and simultaneously serves as a template for reverse transcription. Following translation, the P protein binds to a stem-loop structure of pgRNA and triggers assembly of the core proteins into RNA-containing nucleocapsids. Inside the capsids, composed of 90 or 120 dimers of the core protein, the partially double-stranded genome is synthesized through reverse transcription of the pgRNA [28], [29]. Mature DNA-containing nucleocapsids, formed in the cytoplasm, can then be enclosed by the viral envelope composed of cellular lipids and three viral glycoproteins, the small S, middle M, and large L envelope protein that originate at the endoplasmic reticulum (ER) membrane [30], [31]. Subsequent budding of HBV is supposed to occur at intracellular membranes [30], [31] and involves downstream functions of the ESCRT machinery, as perturbations of ESCRT-III and VPS4 by overexpression of dominant-negative (DN) mutants led to a sequestration of the viral structural proteins into detergent-insoluble membrane structures and to a potent decline in virus release [26]. How HBV gains access to the ESCRT pathway is less clear, but has been implicated to involve putative ESCRT-associated proteins, like the ubiquitin-interacting adaptor γ2-adaptin and the Nedd4 ubiquitin ligase [31]–[34]. Nedd4 interacts with the late domain-like PPAY sequence of the HBV capsid in a productive manner, while γ2-adaptin establishes essential contacts with both the capsid and the envelope [33], [35], [36]. In order to unravel the interplay between HBV and the ESCRT network, we here analyzed the impact of the upstream-acting ESCRT-I and ESCRT-II complexes in HBV maturation and egress.

## Materials and Methods

### Plasmids

For HBV replication in human hepatoma HuH-7 cells, the mammalian episomal expression vector pCEP4ΔCMVΔSV40/1.1xHBV (pHBV) was used. It carries a 1.1x unit length HBV genome (genotype D) in which the viral core/P promoter is preceded by the human metallothionein IIa (hMT) promoter, while the envelope open reading frame is under the transcriptional control of its authentic promoters [37]. The pCEP vector backbone was modified in such that the CMV promoter region and the SV40 polyadenylation signal were deleted. Plasmid pHBVΔC is identical to pHBV with the exception that a stop codon was created at triplet 38 of the core gene to ablate the core expression from the HBV replicon and concomitant virus production. For HBV transcription analyses, a modified version of the HBV replicon plasmid was engineered (pHBVΔHP) in which the foreign hMT promoter region preceding the viral core/P promoter was removed by cloning. For solitary expression of the HBV core protein, plasmid pNI2.C was employed [38]. Full-length cDNA clones encoding human EAP30 (NM_007241) and EAP45 (NM_016075) were obtained from imaGenes (Germany), while a human EAP20 (NM_032353) construct was purchased from Origene. For C-terminal tagging of the ESCRT-II subunits with the FLAG epitope, the corresponding coding regions were PCR-amplified and cloned into p3xFLAG-CMV-14 (Sigma-Aldrich). All constructs were verified by sequencing, and cloning details are available on request. To control FLAG-specific immunoprecipitation reactions, a FLAG-tagged construct encoding the HECT domain of the human ubiquitin ligase Nedd4.1 (NM_006154.2) (amino acids 543 to 897) was used (a gift from Eva Gottwein, University of Heidelberg, Germany).

### siRNAs

To inhibit expression of the ESCRT subunits Tsg101, Vps28, EAP30, and EAP45 the following siRNA duplexes (Sigma-Aldrich) were used: siTsg101#1: CUAUUGAAGACACUAUCUU; siTsg101#2: CUCAAUGCCUUGAAACGAA, siVps28#1: GGCUCCUGGUCCAAUACAA; siVps28#2: GGCUCAGAAAUCAGCUCUA; siEAP30#1: CUUGCAGAGGCCAAGUAUA; EAP30#2: GAAUGGAGGUCUGAUAACU; siEAP45#1: GGAAUAUUGCAGGUGCCUU; siEAP45#2: GGAAUUGGGAAGAGUGCCA. For depletion of EAP20, siGENOME SMARTpool RNAs with the sequences: GUCGAUCCAGAUUGUAUUA, GGGAAACUCAUCUAUCAGU, GCACAAGGCCGAGAUCAUC, and CAGAACAACUCCGUCUUUA were used (Thermo Scientific). A control siRNA with no known homology to mammalian genes was obtained from Qiagen.

### Antibodies

For immunoprecipitation of HBV virions, a mixture of rabbit antibodies against the L and S envelope proteins was used as described [26]. Polyclonal antisera against recombinant native (K45) or denatured (K46) core particles were raised in rabbits [33]. The MA18/7 mouse antibody recognizing the HBV L protein was a gift from K.-H. Heermann (University of Göttingen, Germany). Commercially available antibodies were as follows: mouse anti-β-actin (Sigma-Aldrich), rabbit anti-CD63 (Santa Cruz Biotechnology), mouse anti-EAP20 (VPS25, Santa Cruz Biotechnology), rabbit anti-EAP30 (Santa Cruz Biotechnology), rabbit anti-EAP45 (Santa Cruz Biotechnology), mouse anti-FLAG (Sigma-Aldrich), mouse anti-Tsg101 (Santa Cruz Biotechnology), and rabbit anti-Vps28 (Santa Cruz Biotechnology). Peroxidase-labeled, secondary antibodies were obtained from Dianova, and fluorophor-labeled antibodies were purchased from Molecular Probes.

### Cell Culture and Transfection

The human hepatocellular carcinoma cell line HuH-7 was obtained from the Japanese Collection of Research Bioresources and used throughout. Cells were cultured in Dulbecco’s modified Eagle’s medium supplemented with 10% fetal bovine serum, 5 µg/ml ciprofloxacin, and 1 mM sodium pyruvate. Transfections of cells with plasmid DNAs were performed with LipoFectamine Plus (Invitrogen) as instructed by the supplier. Generally, experiments were performed in 12-well format. For transfection of cells with siRNAs, the LipoFectamine RNAiMAX transfection reagent (Invitrogen) was used. Briefly, cells were transfected with a final concentration of 30 nM siRNA per sample according to the protocol of the supplier. After 48 h, cells were retransfected with plasmid DNA using LipoFectamine Plus (Invitrogen) and harvested after additional 72 h. In the case of kinetic studies, cells were lysed 24, 36, or 48 h after DNA transfection.

### Cell Lysate Preparation and Protein Analyses

For HBV replication in HuH-7 cells, plasmids pHBV or pHBVΔHP were used. After transient expression, cellular supernatants were harvested, and cell lysates were prepared with either detergent-containing lysis buffer or by repetitive freeze-thaw cycles. For lysis with Triton X-100, cells were incubated with 50 mM Tris-HCl, pH 7.5, 150 mM NaCl, 5 mM MgCl_2_, 0.2% Triton X-100 (T-lysis buffer) for 20 min on ice. Thereafter, lysates were centrifuged for 5 min at 15,000×*g* and 4°C and stored at −20°C. Where indicated, cells were lysed by osmotic shock in a hypotonic lysis buffer (10 mM Tris-HCl, pH 7.5, 10 mM NaCl, 1.5 mM MgCl_2_) for 15 minutes on ice and three subsequent freeze-thaw cycles (using liquid nitrogen to freeze and a 37°C water bath to thaw). Lysates were supplemented with NaCl to a final concentration of 150 mM and centrifuged for 30 min at 15,000×*g* and 4°C. To analyze protein expression levels, lysates were assayed by immunoblotting and ELISAs. SDS-PAGE and Western blotting procedures were performed using standard protocols. To probe for the synthesis of the HBV S envelope protein, hepatitis B surface antigen (HBsAg) reactivity of cell lysates was determined with the Murex HBsAg Version 3 kit (Abbott). The biosynthesis of the HBV preCore protein was assayed with the ETI-EBK PLUS ELISA kit (DiaSorin) as instructed by the supplier. To evaluate the presence of damage and toxicity of transfected cells, lactate dehydrogenase (LDH) activity was determined in culture media using a colorimetric quantification assay (Cytotoxicity Detection Kit; Roche Diagnostics).

### Polyethylene Glycol (PEG) Precipitation and Western Blotting of Cytoplasmic Capsids

For concentration of intracellular capsids, they were precipitated with PEG as described [38]. Precipitates were separated by native 1% (w/v) agarose-Tris-acetate-EDTA gel electrophoresis. The gel was blotted by capillary transfer using a nitrocellulose membrane and 10×SSC buffer (1.5 M NaCl, 150 mM sodium citrate, pH 7.0). The membrane was reacted with anti-capsid antibodies using standard techniques.

### Analysis of HBV Replication by Multiplex Real-time PCR

The production of HBV particles was determined by a TaqMan chemistry-based, multiplex real-time PCR. Nucleocapsids and virions were isolated by immunomagnetic separation using PureProteome Protein G Magnetic Beads (Millipore). For precleaning, cellular supernatants and lysates were reacted with non-coated magnetic beads. To precipitate virions, the beads were coupled with the envelope-specific antibodies. For the isolation of nucleocapsids, the beads were coated with capsid-specific antibodies (K45). The immunoprecipitated particles were next subjected to a treatment with 100 U/ml benzonase (Merck) for 4 h at 37°C in the presence of 0.2% Triton X-100 to remove residual plasmid DNA. The viral DNA was isolated with the High Pure Viral Nucleic Acid Kit (Roche Diagnostics) according to the manufacturer’s instructions. PCR analyses were performed with a 7300 Real-Time PCR System and Sequence Detection Software 4.0 (Applied Biosystems). Because the transfected HBV replicon plasmid DNA and progeny virus DNA are genetically identical, two primer/probe sets were designed targeting either the HBV genome (HBV-probe) or the hygromycin resistance gene of the pCEP plasmid backbone (HYG-probe). Progeny viral DNA is amplified by the HBV-primer/probe, whereas plasmid DNA is a target for both primer/probe sets. The subtraction of the values of the HYG-probe from those of the HBV-probe enabled a precise quantification of the viral genome copies. The primer/probe sets were as follows: *HBV-assay*: HBV-F: 5′-TGTCCTCCAACTTGTCCTGGTT-3′, (nucleotide [nt] positions 349 to 370, as referred to the HBV genome, genotype D); HBV-R: 5′-AGGCATAGCAGCAGGATGAAGA-3′, (nt positions 407 to 428); HBV-probe: 5′-FAM-ATCGCTGGATGTGTCTGCGGCGTT-BHQ1–3′. *HYG-assay:* Hyg-F: 5′-AGCGAGAGCCTGACCTATTGCAT-3′; Hyg-R: 5′-AGTTCGGTTTCAGGCAGGTCTT-3′; HYG-probe: 5′-Yakima Yellow-TCCCGCCGTGCACAGGGTGTCACGTT-BHQ1–3′. Each amplification reaction mixture (20 µl) contained 5 µl template DNA, 10 µl Fast Start Universal Probe Master (Roche Diagnostics), 0.4 µl of each primer (10 µM), 0.2 µl of the appropriate probe (10 µM) and 4 µl aqua bidest. The PCR reaction was initiated by a single step at 50°C for two min and 95°C for ten min, followed by 38 cycles at 95°C for 15 sec and 60°C for 60 sec, respectively. All PCR reactions were performed in duplicate and the quantification was based on calibration curves generated by the amplification of a serial dilution of pHBV or pHBVΔHP (1×10^−1^–1×10^6^ fg/µl) for both the HBV-assay and the HYG-assay, respectively.

### Analysis of HBV Replication by Southern Blot

Transfected cells were lysed with the T-lysis buffer as outlined above. The clarified lysate was incubated with 100 U/ml benzonase (Merck) for 4 h at 37°C to remove the transfected plasmid DNAs. DNA was isolated using the High Pure Viral Nucleic Acid Kit (Roche Diagnostics) according to the manufacturer’s instructions and separated in a 1.3% (w/v) agarose-acetate-EDTA gel. After denaturation and neutralization, the DNA was transferred to a positively charged nylon membrane (BrightStar-Plus; Ambion) by capillary blotting for 4 h using 20×SSC (3 M NaCl, 300 mM sodium citrate, pH 7.0) and subsequently crosslinked by UV radiation at 120 mJ/cm^2^. The membrane was hybridized with an *Eco*RI-linearized unit-length HBV genome that was labeled with digoxygenin-dUTP by random priming as instructed by the manufacturer (Roche Diagnostics). The prehybridization, hybridization, and immunological detection reactions were performed according to the instructions of the supplier (DIG High Prime DNA Labeling and Detection Starter Kit II; Roche Diagnostics). Briefly, after prehybridization for 30 min at 48°C in DIG Easy Hyb buffer, the membrane was hybridized overnight at 48°C with the DIG-labeled probe diluted in DIG Easy Hyb buffer at a final concentration of 25 ng/ml. X-ray films (Super RX; Fujifilm) were used for detection.

### mRNA Extraction and Quantification

The mRNA of transfected cells was extracted by using the Dynabeads mRNA DIRECT Kit (Life Technologies), according to the mini scale protocol of the supplier. The mRNA was then treated with 0.4 U/µl RNase-free, recombinant DNase I (Roche Diagnostics), and cDNA synthesis was performed by using the Transcriptor Universal cDNA Master Kit (Roche Diagnostics). For real-time quantitative PCR, the HBV-specific primer/probe set (*HBV-assay*, see above) and a β-actin-specific primer/probe combination as reference were used (*β-actin-assay*: β-actin-F: 5′-TGAAGATCAAGATCATTGCTCCTCC-3′, β-actin-R: 5′-AGAAGCATTTGCGGTGGACGAT-3′, β-actin-Probe: 5′-FAM-TGAGCGCAAGTACTCCGTGTGGATCGG-BHQ1–3′). The PCR was performed as absolute quantification based on calibration curves for both PCR assays, and HBV-quantities were related to β-actin-quantities of the same sample. The HBV primers target the 3.5-kb pgRNA, the 2.4- and 2.1-kb envelope-specific transcripts, but not the 0.7-kb X-specific mRNA.

### HBV pgRNA Extraction and Quantification

Transfected cells were lysed by freeze-thaw cycles and intracellular capsids were isolated with immunomagnetic beads coated with K45-antibodies. Beforehand, an aliquot of the lysate was removed for RT-qPCR of the β-actin mRNA as a reference. The encapsidated pgRNA and total cell mRNA were isolated with TRIzol Reagent (Life Technologies), according to the protocol of the manufacturer. Prior to RNA precipitation, 10 µg glycogen (Peqlab) was added per sample as carrier. The isolated RNA was treated with DNase I, reverse transcribed, and quantified, exactly as outlined above.

### Coimmunoprecipitation Assay

To probe for complex formation, cells were lysed with a 2% solution of the non-denaturating detergent CHAPS {3-[(3-cholamidopropyl)-dimethylammonio]-1-propanesulfonate}-HBS (50 mM Hepes-KCl pH 7.5/200 mM NaCl), 20 mM N-ethylmalemide, supplemented with 1 × protease inhibitor mixture (Serva) for 20 min on ice. After centrifugation, lysates were immediately subjected to immunoprecipitation using tosyl-activated, superparamagnetic polystyrene beads (Dynabeads Sheep anti-rabbit IgG; DYNAL) that had been precoated with the anti-core antibody K45 as described [33]. After incubation for 3 h at 4°C with agitation, the immune complexes were washed three times with 0.5% CHAPS/HBS, and once with phosphate-buffered saline (PBS) prior to SDS-PAGE and immunoblotting.

### Fluorescence Microscopy

For immunostaining, cells grown on cover-slips were fixed and permeabilized with ice-cold methanol containing 2 mM EGTA. Cells were blocked in PBS containing 1% bovine serum albumin, incubated with the indicated primary antibodies for 1 h at 37°C, rinsed with PBS, and then incubated with AlexaFluor-conjugated secondary antibodies for 1 h at 37°C. DNA was stained with Hoechst 33342 (Sigma-Aldrich). Z-stack images were acquired separately for each channel using a Zeiss Axiovert 200 M microscope equipped with a Plan-Apochromat 100× (1.4 NA) and a Zeiss Axiocam digital camera. Axiovision software 4.7.1 was used for merging pictures. Z-stack images were optically deconvoluted using the software supplied by Zeiss, and the degree of colocalization was measured with the Axiovision “colocalization” module. Tiffs were assembled into figures using Photoshop CS2 (Adobe).

## Results

### ESCRT-I is Not Essential for HBV Production

**Figure 1:**
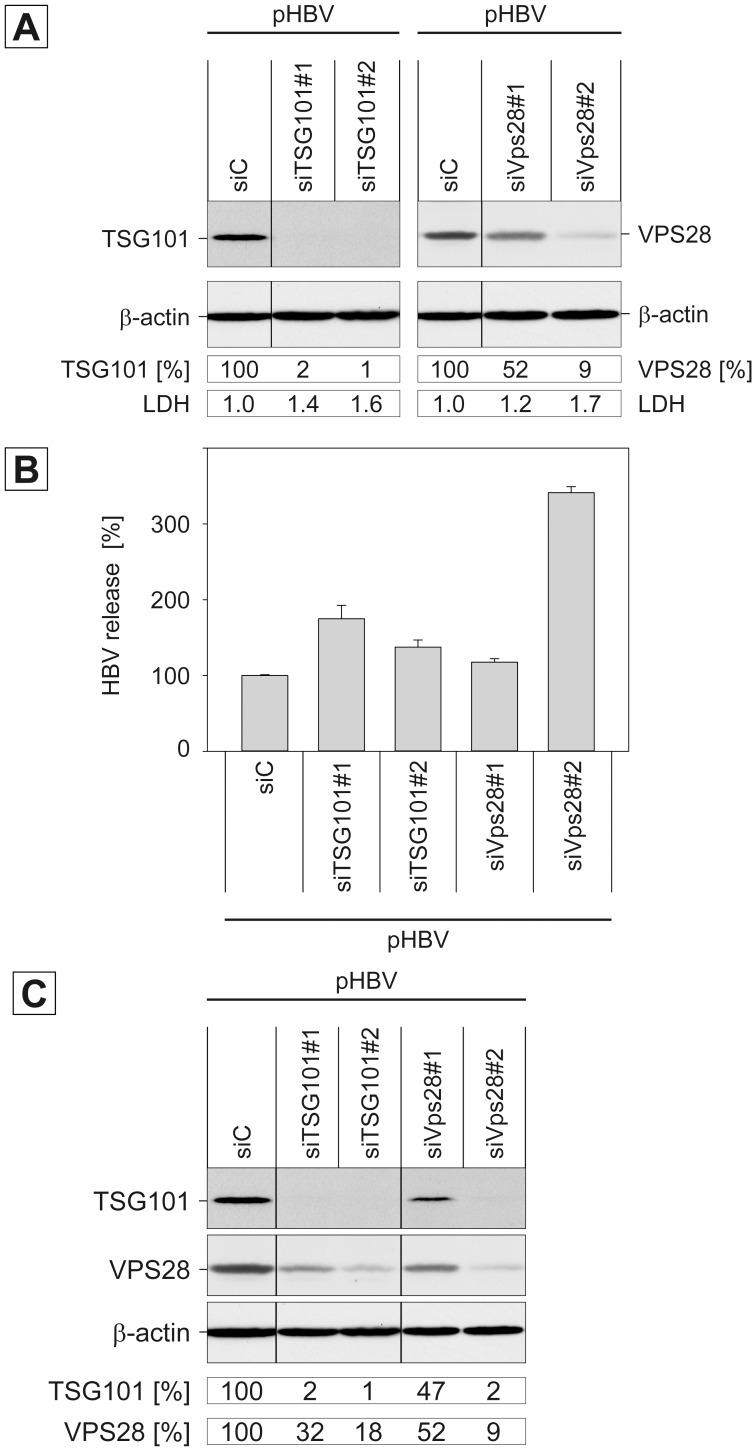
Impact of ESCRT-I subunits TSG101 and VPS28 on HBV production. **A.** HuH-7 cells were treated with control siRNA (siC) or two individual siRNAs targeting TSG101 or VPS28. Cells were subsequently transfected with the pHBV replicon. The knockdown efficacy of the siRNAs was analyzed by Western blotting of cellular lysates using TSG101- or VPS28-specific antibodies. Uniformity of sample loading was confirmed by probing the lysates with anti-β-actin antibodies. Relative protein expression values were determined by densitometric analysis and demonstrated in percent amount relative to control cells. To probe for cytotoxicity, cellular supernatants were assayed for LDH activity and indicated as factors of increase relative to control cells. **B.** The number of virions released into the cell culture supernatants were quantitated by real-time PCR of the viral genomes and demonstrated in percent amount relative to control siRNA-treated cells. Error bars indicate the standard deviations from the mean of four experiments measured in duplicates. **C.** Co-depletion effects of ESCRT-I-specific siRNAs. Control siRNA or the two siRNAs targeting either TSG101 or VPS28 were introduced into HuH-7 cells prior to transfection with pHBV. Cell lysates were prepared and subjected to Western blotting using anti-TSG101, anti-VPS28, and anti-ß-actin antibodies. TSG101- and VPS28-specific band intensities were quantitated and demonstrated in percent amount relative to siC-transfected cells.

The heterotetrameric ESCRT-I complex is composed of a single copy of the unique TSG101 subunit and single copies of one of the different isoforms of VPS28 (two variants, A and B), VPS37 (four isoforms, A–D), and MVB12 (three isoforms, MVB12A, MVB12B, UBAP1) and links membrane-specific adaptors and MVB protein cargoes to the downstream ESCRT machinery [1]–[3], [5]. To analyze whether ESCRT-I complex partners are involved in HBV morphogenesis, we performed a siRNA-mediated knockdown of TSG101 and VPS28 in HBV-replicating HuH-7 liver cell lines using two individual siRNA duplexes per target protein. Of note, the VPS28-specific siRNA duplexes target both VPS28 variants. After 48 h, cells were retransfected with the plasmid-based pHBV replicon, and cell lysates and supernatants were harvested after additional 72 h. To probe for the knockdown efficacy, lysates were subjected to Western blotting using TSG101- and VPS28-specific antibodies. For quantification, band intensities were analyzed by densitometry with endogenous β-actin levels serving as a reference. In the case of TSG101, both siRNAs almost completely blocked its expression, while for VPS28 silencing effects varied between 9% (±8.2 SD, *n* = 3) for the siVps28#2 duplexes to 52% (±7.8, *n* = 3) for the Vps28#1 duplexes (Fig. 1A). Cytotoxicity assays of supernatants did not reveal significant evidence of cell damage, indicating that the applied RNA interference strategy is not harmful for the cells. The production and egress of HBV particles was determined by immunocapture with envelope-specific antibodies followed by particle disruption and real-time PCR measurement of the number of HBV genomes. As shown in Fig. 1B, the perturbation of ESCRT-I functions did not decrease but rather increase HBV release. All siRNAs, targeting either TSG101 or VPS28, reproducibly evoked a stimulation of virus export in the range of 0.2- to 3.5-fold as compared to control siRNA-treated cells. Intriguingly, the effective siVps28#2-induced depletion of VPS28 led to a particular robust increase in HBV release (Fig. 1A and B). Since VPS28 is a direct interaction partner of the ESCRT-II subunit EAP45 [7], the lack of VPS28 might augment the availability of EAP45.

The knockdown of individual subunits of protein complexes often triggers the breakdown of the entire complex likely because it loses its integrity [10], [11], [39], [40]. In order to analyze the properties of ESCRT-I, HuH-7 cells were treated with the TSG101- and VPS28-specific siRNAs and assessed for cross-depletion. As shown in Fig. 1C, the siRNAs reduced the expression of their targets as compared to control cells. Importantly, depletion of TSG101 decreases the level of VPS28 and vice versa. The degree of co-depletion reflected a tight relationship, as siRNAs with an excellent, target-specific efficacy also showed a high competence to down-regulate the ESCRT-I partner protein (Fig. 1C). From these data we hypothesize that TSG101 and VPS28 are important to stabilize the whole ESCRT-I complex. Accordingly, we assume that the loss of ESCRT-I rather than its individual subunits is likely responsible for the enhancement of HBV egress. Based on these findings we did not further inspect the involvement of VPS37 and MVB12 in HBV production, whose depletion is impeded due to the existence of their multiple isoforms.

### ESCRT-II is Essential for HBV Production

**Figure 2:**
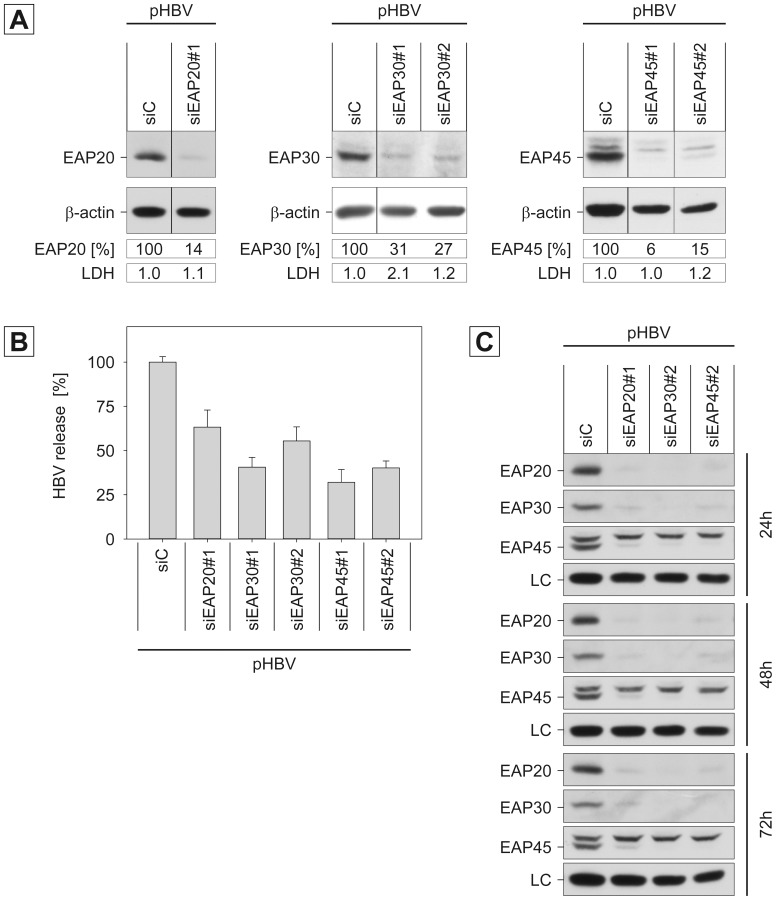
Impact of ESCRT-II subunits EAP20, EAP30, and EAP45 on HBV production. **A.** HuH-7 cells were treated with control siRNA (siC), a siRNA pool targeting EAP20, or two different siRNA duplexes directed against EAP30 or EAP45. After 48 h, cells were retransfected with pHBV, and lysates and supernatants were harvested 72 h later. To probe for the efficiency of the knockdowns, lysates were immunoblotted with antibodies against EAP20, EAP30, and EAP45. Identical sample loading was assessed by anti-β-actin Western blotting, and relative protein expression values were determined by densitometric analysis and demonstrated in percent amount relative to control cells. To probe for cell lysis, supernatants were assayed for LDH activity. **B.** Virions released into the cellular supernatants were quantified by real-time PCR of the HBV genomes. Error bars indicate the standard deviations from the mean of four experiments measured in duplicates. **C.** Kinetic and co-depletion effects of the ESCRT-II-specific siRNAs. Cells were treated with the indicated siRNAs for 48 h, transfected with pHBV and lysed after 24, 48, or 72 h DNA transfection. RNAi effects on the expression of EAP20, EAP30, and EAP45 were analyzed by specific immunoblotting. A non-specific band stained by the antisera served as a control for identical gel loading (LC).

Next, we analyzed the role of the heterotetrameric ESCRT-II complex in HBV morphogenesis. ESCRT-II contains single copies of EAP45 and EAP30 as well as two copies of EAP20 and can bridge the ESCRT-I with the ESCRT-III complexes via interactions with the ESCRT-I subunit VPS28 and the ESCRT-III component CHMP6 [2], [3], [41]. In yeast, ESCRT-II activity is essential for MVB cargo sorting. Whether ESCRT-II may perform a similar role at the mammalian MVB has not yet been fully disclosed, as there are inconsistent reports about the requirement of ESCRT-II for ligand-dependent epidermal growth factor receptor degradation [8], [10], [11]. Again, two different siRNAs were used to silence the ESCRT-II subunits EAP30 and EAP45 in HBV-replicating HuH-7 cells. For the knockdown of EAP20, a pool of four different siRNAs was applied. Cell lysates and supernatants were investigated as above and extracellular progeny HBV genomes were quantitated by PCR. As shown by EAP20-, EAP30-, and EAP-45-specific immunoblotting, the siRNAs effectively reduced the expression of their targets as compared to control siRNA-treated cells (Fig. 2A). Neither knockdown affected overall cell viability and/or protein expression, as evidenced by lactate dehydrogenase (LDH) activity assays and β-actin immunoblotting (Fig. 2A). However, unlike ESCRT-I, the perturbation of ESCRT-II activities led to a substantial decline of extracellular virions (Fig. 2B). To ascertain the RNA interference efficiency over the time course, we kinetically analyzed the silencing effects of the ESCRT-II-specific siRNAs. Therefore, cells were lysed 24 h, 48 h, and 72 h after transfection with the HBV replicon and subjected to EAP20-, EAP30-, and EAP-45-specific immunoblotting. As shown in Fig. 2C, an effective knockdown of either ESCRT-II subunit was observed at each measured time point. To inspect the impact of silencing of individual ESCRT-II subunits on the protein level of their complex partners, the same samples were tested for co-depletion. Each siRNA not only down-regulated its specific target but also the complex partner proteins (Fig. 2C). Since these data indicate that the ESCRT-II complex loses its compactness upon depletion of one subunit, the entire ESCRT-II complex likely contributes to HBV release.

### Role of ESCRT-II in HBV Maturation

**Figure 3:**
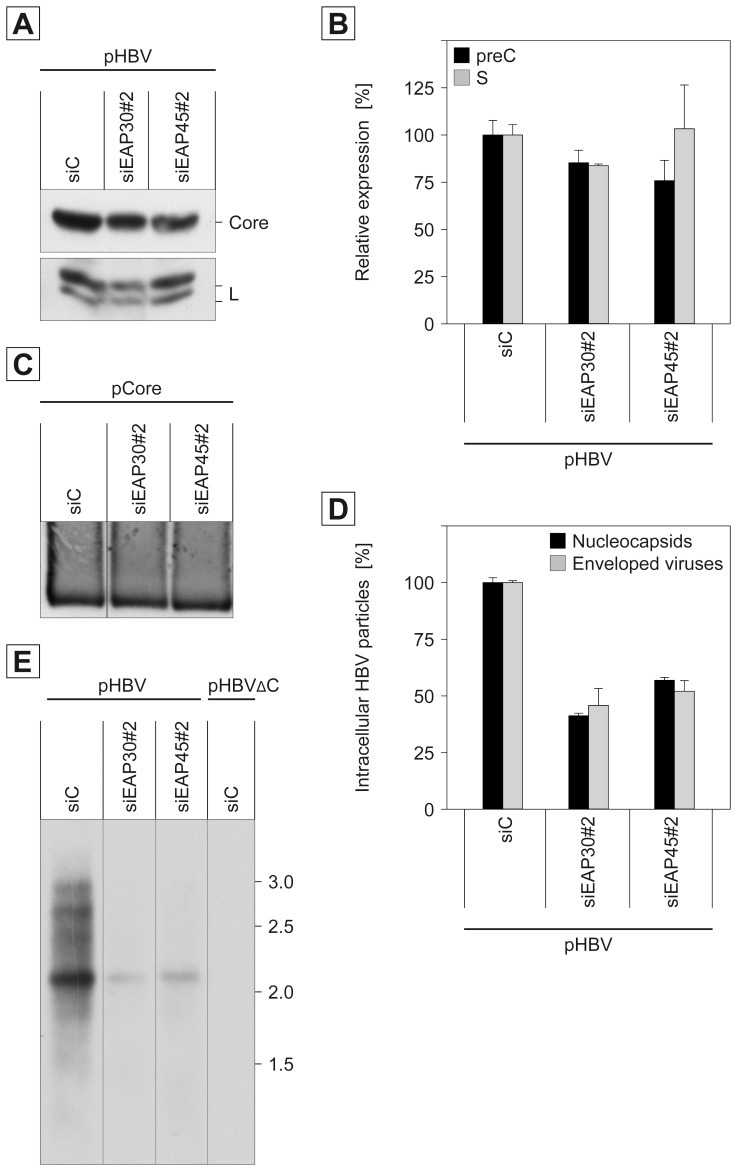
Impact of ESCRT-II subunits EAP30 and EAP45 on HBV maturation. HuH-7 cells were treated with control siRNA (siC) or the siRNA duplexes siEAP30#2 or siEAP45#2. After 48 h, cells were retransfected with pHBV, and cell lysates and supernatants were harvested 72 h later. **A.** Lysates were prepared with detergent and assayed for the synthesis of the HBV core and L envelope (L) proteins by specific Western blotting. **B.** Lysates were probed for the synthesis of the secretory precore (preC) and S envelope (S) proteins by specific ELISAs. Intracellular amounts of preC and S are expressed as mean units of optical density at 492 nm (*n = *2) and demonstrated in percent amount relative to siC-treated cells. **C.** Following treatment with siC, siEAP30#2, or siEAP45#2, cells were transfected with the vector pCore encoding solely the HBV core protein. Cytoplasmic capsids were concentrated by PEG precipitation and separated in a native agarose gel, blotted, and detected with anti-capsid antibodies. **D.** Cells were treated with the indicated siRNAs followed by transfection with pHBV. Cells were lysed by repetitive freeze-thaw cycles, and intracellular nucleocapsids and enveloped virions were precipitated with capsid- or envelope-specific antibodies, respectively, and assayed by quantitative real time PCR. Error bars indicate the standard deviations from the mean of two experiments measured in duplicates. **E.** Cells were transfected exactly as outlined in **D**. For a control, a replication-defective pHBVΔC replicon was included in the Southern blot analysis. Intracellular viral DNA was isolated and processed by Southern blotting using a digoxigenin-dUTP-labeled HBV-specific probe. Numbers to the right refer to marker DNA in kb.

To gain insights into the mechanistic action of ESCRT-II, we focused on different steps accompanying HBV maturation. Since single EAP30- and EAP45-specific siRNA duplexes are capable to down-regulate the ESCRT-II complex partner proteins (see Fig. 2), the continuing experiments were performed with the siEAP30#2 and siEAP45#2 reagents. HuH-7 cells were transfected with these or control siRNA duplexes prior to transfection with the pHBV replicon. Cell lysates, prepared with the detergent Triton X-100, were then investigated for the expression and stability profiles of the viral core and envelope proteins using Western blotting and ELISA analyses. The intracellular steady-state levels of the L envelope protein, synthesized in non-glycosylated p39 and single-glycosylated gp42 forms, and the core protein were unaffected, irrespective of whether EAP30 or EAP45 were depleted or not (Fig. 3A). Consistent with this, no significant decline in the synthesis and stability of the S envelope protein or the secretory preCore protein could be detected upon ablation of EAP30 and EAP45 (Fig. 3B). Hence, the inactivation of ESCRT-II does not affect the supply of the viral structural proteins. Next, we asked whether dysfunctional ESCRT-II might interfere with the capsid assembly reaction. During this process, monomeric core proteins rapidly form dimers that package the viral pgRNA together with the bound P protein and assemble into icosahedral nucleocapsids [28], [29]. Notably, however, authentic capsids can be formed even in the absence of the HBV pgRNA/P complex [28], [31], [38], [42], [43]. To probe for capsid assembly, HuH-7 cells were treated with the ESCRT-II-specific siRNAs prior to transfection with the core-encoding plasmid pNI2.C. Lysates were examined for assembled capsids by native agarose gel electrophoresis and Western blotting with anti-capsid antibodies. This analysis revealed no differences in the signal intensities of control, EAP30, or EAP45 siRNA-treated cells (Fig. 3C), indicating that ESCRT-II is dispensable for capsid formation. In order to assess whether the loss of ESCRT-II may affect the nucleocapsid assembly and/or the nucleocapsid envelopment reaction, ESCRT-II-depleted cells were transfected with the pHBV replicon and lysates were prepared by repetitive freeze-thaw cycles. This protocol was chosen in order to avoid the use of detergents that would disrupt the envelope of intracellular virions. Nucleocapsids and enveloped virions, present in cell lysates, were then precipitated with capsid- or envelope-specific antibodies, respectively, and assayed by real time PCR. Against expectation, this analysis reproducibly revealed that the depletion of EAP30 and EAP45 markedly decreased the amount of intracellular nucleocapsids, i.e. the level of nucleocapsid-associated HBV genomic DNA (Fig. 3D). Because only mature DNA-containing nucleocapsids are competent for envelopment [30], [31], [44], [45], the amount of intracellular enveloped nucleocapsids was comparatively and belike consequently reduced upon ESCRT-II deficiency (Fig. 3D). Together, these results demonstrate that ESCRT-II plays a functional role in the formation of mature HBV nucleocapsids rather than in the final budding reaction. Of note, the phenotype provoked by the absence of ESCRT-II is different from those observed upon overexpression of DN versions of ESCRT-III or VPS4 in HBV-replicating cells [25]–[27]. The DN ESCRT-III/VPS4 mutants entrapped the viral core and envelope proteins in detergent-insoluble membrane structures that resembled aberrant MVB compartments. Under those conditions, the egress of HBV but not the formation of replication-active nucleocapsids was blocked provided that the cells were lysed with a denaturing detergent [26].

To obtain further evidence for the action of ESCRT-II in HBV nucleocapsid formation and/or stability, we conducted Southern blot analysis. This technique enables to detect the final HBV genome (i.e. the partially double-stranded DNA) as well as replicative intermediates occurring during reverse transcription. Cells were transfected with the siRNA duplexes and pHBV as above, and viral DNA was extracted from the cell lysates. An HBV replicon that is defective in replication due to an ablation of core protein expression (pHBVΔC) was included as a control. As shown in Fig. 3E, HBV-specific bands could be detected in pHBV-transfected cells but not in pHBVΔC-transfected cells, indicating that the signals were derived from progeny DNA rather than from the transfected HBV plasmid DNA. In siControl-treated cells, pHBV supported the synthesis of different species of HBV replication intermediates, with predominance of single-stranded DNA and growing strands as well as some partially double-stranded DNA (Fig. 3E). The depletion of EAP30 or EAP45 diminished the amount of all DNA species (Fig. 3E), indicative for an impairment of the formation of replication-active nucleocapsids.

### Role of ESCRT-II in HBV Genome Maturation

**Figure 4:**
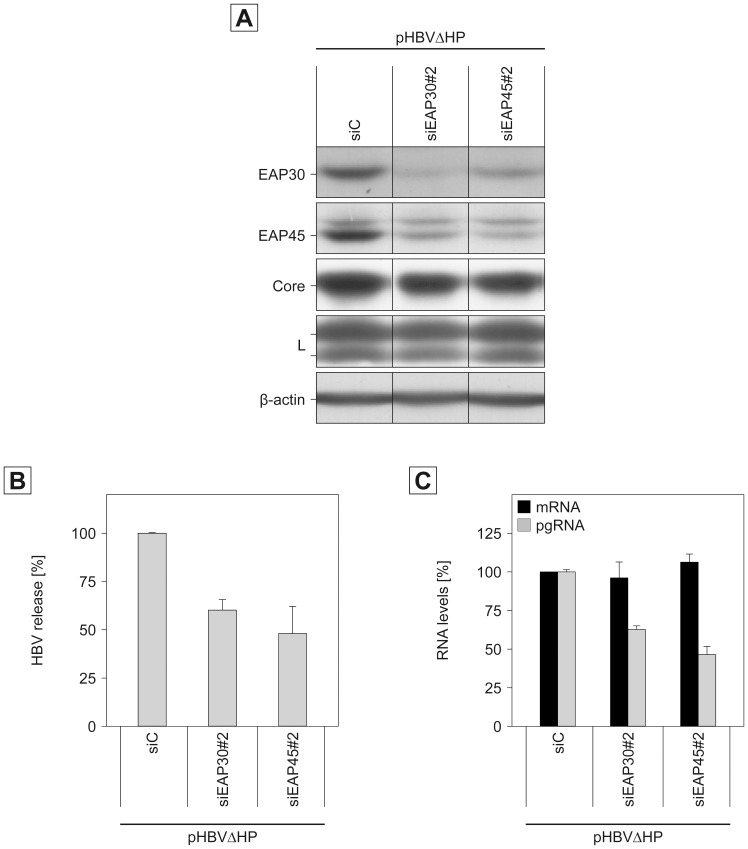
Impact of ESCRT-II subunits EAP30 and EAP45 on HBV genome maturation. HuH-7 cells treated with control siRNA (siC) or siRNAs targeting EAP30 or EAP45 were retransfected with the modified pHBVΔHP replicon devoid of foreign promoter elements. **A.** Lysates were probed with antibodies to EAP30, EAP45, Core, L, and β-actin as indicated. **B.** Virions released into the cellular supernatants were quantified by real-time PCR of the HBV genomes. Error bars indicate the standard deviations from the mean of two experiments measured in duplicates. **C.** For quantitative reverse transcription-PCR (qRT-PCR), total mRNAs were isolated, reverse transcribed and used for PCR reactions. To measure HBV pgRNA packaged into intracellular capsids, lysates were subjected to a capsid-specific immunoprecipitation prior to RNA isolation and qRT-PCR. Error bars indicate the standard deviations from the mean of two experiments measured in duplicates.

Apart from their action in the ESCRT network, subunits of the ESCRT-II complex have been implicated to play a role in transcriptional regulation. In the nucleus, EAP30 and EAP45 interact with the ELL complex, an elongation factor associated with RNA polymerase II [46], hence the name EAP (ELL-associated protein). Therefore, we reasoned that the decrease of nucleocapsid-associated HBV genomic DNA upon ESCRT-II perturbation might be due to alterations in HBV transcription. The pHBV replicon used thus far contains a 1.1× unit length HBV genome in which the heterogeneous hMT promoter precedes the viral core/P promoter [37]. Because this feature might hamper transcriptional analyses, a modified HBV replicon construct (pHBVΔHP) with a deletion of the hMT promoter element was used for these experiments. For functional characterization of the modified replicon construct, HuH-7 cells were treated with control-, EAP30-, or EAP45-specific siRNA duplexes prior to transfection with pHBVΔHP. Western blot analyses of lysates with EAP-30 and EAP-45-specific antibodies confirmed the depletion efficacy of the used siRNAs (Fig. 4A). Consistent with the aforementioned results, the knockdown of ESCRT-II had no effect on the expression and stability profile of the core and L proteins (Fig. 4A), while the number of extracellular progeny virions dropped down up to ∼50% in ESCRT-II-depleted cells (Fig. 4B). We took these data as proof for the functionality of pHBVΔHP.

To study HBV transcription in ESCRT-II depleted cells, total mRNA was extracted and measured by quantitative reverse transcription PCR using HBV-specific primers. As would be expected from the results of the protein expression studies, the functional ablation of EAP30 or EAP45 did not impair the synthesis and stability of HBV-specific mRNAs, indicating that ESCRT-II is not involved in HBV transcription per se. Following transcription and nuclear export, the HBV pgRNA is next packaged into core assemblies in which the reverse transcription reaction takes place. To analyze whether EAP30 or EAP45 may interfere with events accompanying pgRNA trafficking and/or packaging, we focused on the encapsidated HBV transcripts. Therefore, cellular lysates were immunoprecipitated with capsid-specific antibodies prior to RNA extraction and quantitative reverse transcription PCR. Thereby, we observed that the knockdown of EAP30 and EAP45 significantly reduced the pgRNA levels within the nucleocapsids (Fig. 4C). Importantly, the decrease of the encapsidated pgRNA pools closely reflected the declines in extracellular virion levels (see Fig. 4B) imposed by dysfunctional EAP30 and EAP45. Collectively, we conclude from these results that ESCRT-II directs steps involved in the formation and/or stability of replication-competent HBV nucleocapsids.

### ESCRT-II Interacts and Colocalizes with HBV Core

**Figure 5:**
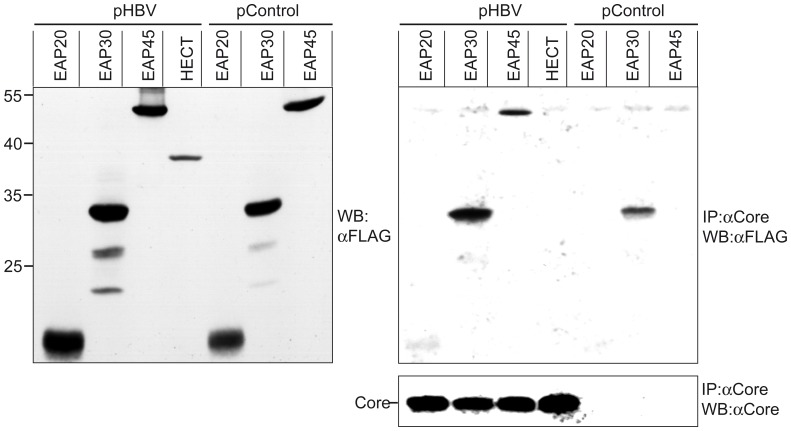
HBV Core interacts with ESCRT-II subunits. HuH-7 cells were cotransfected with FLAG-tagged EAP20, EAP30, or EAP45 together with pHBV or empty plasmid DNA (pControl) at a 1∶1 DNA ratio. As a control, pHBV was cotransfected with a FLAG-tagged construct derived from Nedd4.1 (HECT). Synthesis of the EAP and HECT constructs is shown by immunoblotting of lysates with anti-FLAG antibodies (left). Numbers to the left of the panel refer to molecular weight standards in kDa. For coimmunoprecipitation (IP), lysates were incubated with anti-core antibodies before Western blotting (WB) with the FLAG-specific antibody (top right). The same blot was stripped and reacted with anti-core antibodies (bottom right). The experiment was repeated three times and a representative image is shown.

These findings raised the question how the nucleocapsid may engage ESCRT-II. To address this issue, we analyzed whether the nucleocapsid-forming core protein may physically interact with ESCRT-II. Because the polyclonal antisera against EAP30 and EAP45 proved to be less suitable for immunopecipitation and immunofluorescence studies, we constructed expression vectors encoding human EAP20, EAP30, or EAP45 fused with C-terminal FLAG-tags. The vectors were cotransfected with either empty plasmid DNA or pHBV at a 1∶1 DNA ratio into HuH-7 cells. For control, pHBV was transfected together with a FLAG-tagged construct encoding the HECT domain of the ubiquitin ligase Nedd4.1 that does not interact with core (J. T. Stieler and R. Prange, unpublished observation). Cell extracts were prepared with the mild detergent CHAPS after 72 h, and FLAG-specific immunoblotting showed that EAP20, EAP30, and EAP45 were efficiently synthesized and appeared in about 20, 30, and 45 kDa (plus the 2.5 kDa FLAG-tag) forms, respectively, in accord with their calculated molecular masses (Fig. 5). In the case of EAP30, faster migrating forms were found in addition to the full-length protein that might represent N-terminally degraded polypeptide chains and/or alternative translational initiation products. Lysates were next subjected to immunoprecipitation with anti-core antibodies, and the immune complexes were examined by FLAG-specific immunoblotting. Thereafter, the blot was stripped and probed with core-specific antibodies to verify core precipitation. As shown in Fig. 5, the core protein could indeed coprecipitate each ESCRT-II subunit, albeit with different degrees. Although a portion of EAP30 was nonspecifically precipitated, the subtraction of this portion from the core-specific precipitation revealed a pronounced interaction between core and EAP30. Aside, core specifically brought down EAP45, while the amount of coprecipitated EAP20 was low but repeatedly obtained in independent experiments. In case of the FLAG-tagged HECT polypeptide, included as a negative control, no coprecipitation could be observed even after longer blot exposure (Fig. 5). Previous studies had shown that ectopically expressed, epitope-tagged ESCRT-II subunits interact with each other and even with their endogenous complex partners [8], [10], [11], [40]. Hence, the immunocapture analysis clearly revealed an association of core with ESCRT-II in the order EAP30 > EAP45 > EAP20 but did not ascertain whether core may interact with all or individual ESCRT-II subunits. The comparatively weak interaction between core and EAP20 might be due to multiple complex partners of EAP20 as it pairs not only with its ESCRT-II partners but also with the ESCRT-III subunit CHMP6 [8], [9]. Hence, little unoccupied EAP20 might be available for binding to core.

**Figure 6:**
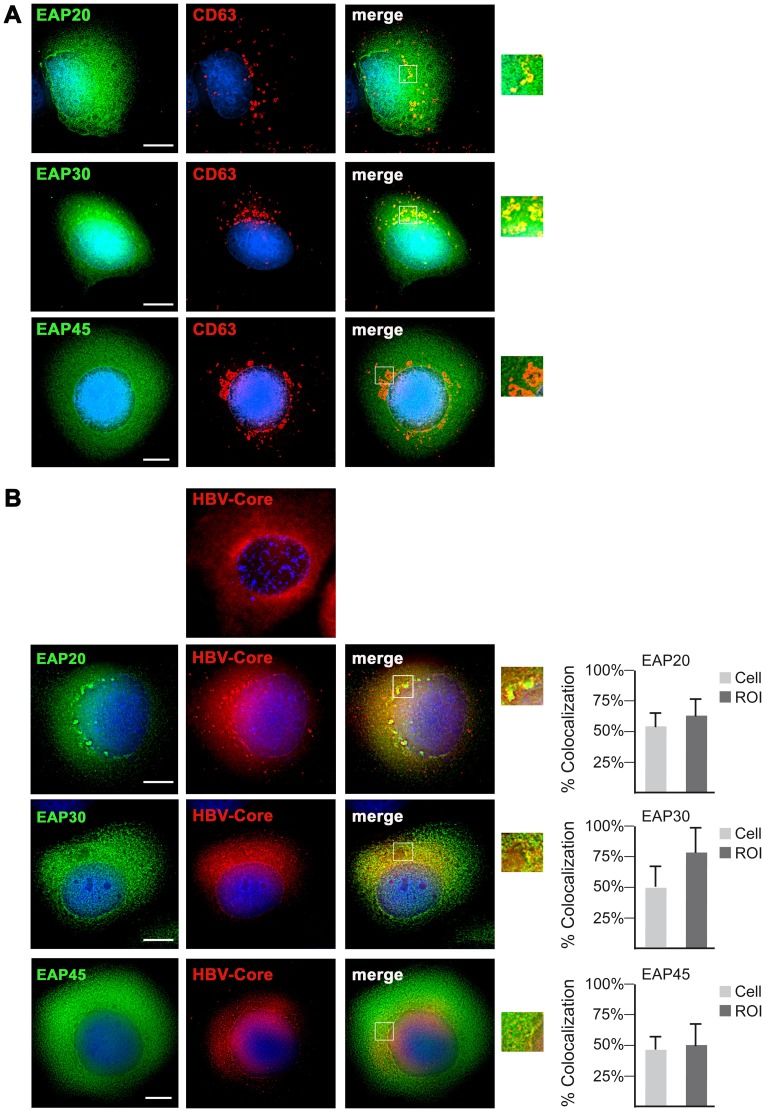
HBV Core colocalizes with ESCRT-II subunits. **A.** HuH-7 cells were transfected with FLAG-tagged EAP20, EAP30, or EAP45 and immunostained with mouse anti-FLAG and rabbit anti-CD63 antibodies. After staining with secondary antibodies, cells were analyzed by deconvolution fluorescence microscopy. The staining pattern of the EAP constructs is shown in green, and the fluorescent signal of CD63 is in red. The overlays of the fluorescence patterns are shown in the right column with yellow color indicating colocalization. Insets in these images display enlarged sections that are shown in the very right column. DNA staining is shown in blue. Bar, 10 µm. **B.** Cells were transfected with pHBV alone or FLAG-tagged EAP20, EAP30, or EAP45 and costained with rabbit anti-core (red) and mouse anti-FLAG (green) antibodies. Colocalization between ESCRT-II subunits and core is indicated in yellow in the right column. Outlined areas of these images are shown at larger magnification. DNA staining is shown in blue. Bar, 10 µm. The degree of colocalization was estimated using the Axiovision image analysis software (Zeiss). Ten cells from three independent transfection experiments were quantitated for the degree of overlap (Cell) or spatial pattern of overlap by analyzing five regions of interest (ROI) per cell. The average of colocalization is plotted in the right graphs.

To corroborate these findings, we performed deconvolution immunofluorescence microscopy to visualize the distribution of the Flag-tagged ESCRT-II subunits in the absence or presence of an ongoing HBV replication. Upon individual transfection, EAP20, EAP30, and EAP45 all were distributed throughout the cytoplasm with some nuclear localization (Fig. 6A) that is consistent with published data [11], [40]. When cells were costained with antibodies against CD63, a marker for late endosomes and MVBs, EAP20-FLAG and EAP30-FLAG, but not EAP45-FLAG, showed a substantial overlapping localization with CD63 (Fig. 6A). This suggests that overexpressed EAP20-FLAG and EAP30-Flag are functional and localize to a subset of MVBs or late endosomes, likely due to complex formation with their endogenous partner proteins. To account for the phenotype of EAP45-FLAG, the addition of the highly negatively charged FLAG tag might affect its charge distribution and/or structure. Since EAP45 contains a positively charged lipid-binding pocket that mediates association with phosphoinositides [47], the artificial FLAG tag might compromise the binding to late endosomal membranes. In cells transfected with the pHBV replicon alone, the HBV core yielded its typical cytoplasmic staining with some enrichment in juxtanuclear regions (Fig. 6B). The distribution of core did not grossly changed upon cotransfection with either ESCRT-II construct save for some enrichment of core in dot-like structures in EAP20-FLAG-transfected cells. The overlay of the fluorescence patterns revealed that core partly colocalized with each ESCRT-II subunit (Fig. 6B). For quantitation, the degree of entire overlap and the spatial pattern of overlap were examined. Thereby, we found that EAP30-FLAG showed the highest extent of colocalization with core, while EAP20-FLAG-expressing cells displayed about 65% colocalization with core in regions of interest (Fig. 6B). EAP45-FLAG exhibited the lowest degree of colocalization, possibly due to its limited capacity to associate with membranes and/or to pair with endogenous ESCRT-II partner proteins and/or (see Fig. 6A). Combining the biochemical and imaging data, we conclude that the HBV core interacts with ESCRT-II.

## Discussion

Previous work had shown that HBV budding involves late scission functions provided by ESCRT-III and VPS4 [25]–[27]. By analyzing the impact of the upstream-acting ESCRT-I and ESCRT-II complexes in HBV egress, here we report that an RNAi-mediated depletion of the ESCRT-I subunits TSG101 or VPS28 did not block, but enhance virus release. By contrast, the suppression of the ESCRT-II components EAP20, EAP30 or EAP45 inhibited virus production. Consistent with several reports [10], [11], [38]–[40], we observed that the knockdown of a single subunit of either ESCRT-I or ESCRT-II decreases the expression level of complex partner proteins, likely because the complexes lose their integrity and stability. Hence, the observed effects on HBV biology appear to be due to the loss of the genuine ESCRT-I and ESCRT-II complexes.

Since the HBV structural proteins lack a P(S/T)AP-like late domain motif, it was less surprising to find that TSG101/ESCRT-I is dispensable for HBV egress. Rather, the inactivation of ESCRT-I led to a slight, but reproducible increase in virus production. To account for this, we consider that processes depending on the entire modular ESCRT cascade may be transiently inactivated. As a consequence, more functional ESCRT-II – and in turn ESCRT-III/VPS4– would be available to assist in HBV maturation and release. Similar results have been revealed for the TSG101/ESCRT-I-dependent human immunodeficiency virus type 1 (HIV-1) that can reportedly bud from cells lacking functional EAP20 [8], [23]. The depletion of EAP20 or the overexpression of a fragment of Sprouty2, an EAP20-interacting protein that disrupts ESCRT-I interaction with ESCRT-II, increase HIV-1 Gag release [8], [48], likely due to the higher availability of ESCRT-I. The TSG101/ESCRT-I independence of HBV appears to be in conflict with a very recent report suggesting a role for Tsg101 in the HBV life cycle [49]. In this report, human α-taxilin was identified as a specific binding partner of the HBV L envelope protein and as an important factor for virus release. Because α-taxilin is also able to interact with Tsg101, these experiments have been interpreted as indication that α-taxilin may act as an adaptor to link the ESCRT machinery to the HBV envelope proteins [48]. While such interactions may contribute to HBV production, the results of our RNAi experiments show that Tsg101 is not an essential factor for virus release.

Enveloped virus budding differentially employs ESCRT functions depending on the particular viral late domain. ESCRT-dependent virus budding necessarily requires the functions of ESCRT-III and VPS4 but rarely engages ESCRT-II [8], [14], [15], [23], [24]. Functional studies utilizing the overexpression of DN forms of the three ESCRT-II subunits or the depletion of EAP45 had shown that (L)YPX_n_L-dependent viruses, like the equine infectious anemia virus, do not require ESCRT-II for budding [22], [24]. Rather, these viruses appear to bypass ESCRT-I and –II by engaging Alix to recruit the downstream scission machinery [21]. Similar results were reported for the P(S/T)AP-dependent HIV-1 that successfully buds in EAP20-depleted cells [8], [19], [23]. More recent studies, however, have challenged the apparent lack of ESCRT-II requirement in HIV-1 budding (i) by demonstrating a role for EAP30/ESCRT-II in HIV-1 genomic RNA trafficking and virus production [50], and (ii) by establishing that ESCRT-II is an integral part of the HIV-1 Gag budding process upon *in vitro* assembly studies [51]. In the case of PPXY-encoding viruses, there is a report that EAP20/ESCRT-II is required for budding of avian sarcoma virus (ASV) Gag particles [23]. Intriguingly, in analogy to our findings for HBV, the egress of ASV Gag particles is also independent of ESCRT-I [23], [52]. Among PPXY-encoding viruses, HBV and ASV are unique in that they contain a single known late domain [23], [33], [53]. Other viruses of this class frequently contain the PPXY motif in combination with further late domains [14]–[18]. Hence, it is tempting to speculate that the sole PPXY motifs of HBV and ASV may direct the preferential use of ESCRT-II.

In addition, the type of membrane used by enveloped viruses for assembly and budding may specify their entry portal into the ESCRT cascade. Viruses that rely on the function of TSG101 or Alix are known to mainly bud from the plasma membrane of infected cells [14], [16], [17]. The budding site of HBV has not been unequivocally established but appears to involve ER/Golgi and/or endosomal structures as conveyed by electron and fluorescence microscopy [26], [30], [54]. Similarly, as evidenced by a lipid staining strategy, ASV Gag assembly occurs at endosome-derived membranes rather than at the plasma membrane [17], [52]. Within the ESCRT pathway, there are three phosphoinositide-recognition domains, including the FYVE domain of ESCRT-0, the GLUE (GRAM-like ubiquitin-binding in EAP45) domain of ESCRT-II, and the CHMP3 subunit of ESCRT-III that differ in their lipid preferences [4], [7], [55]. Because the GLUE domain of ESCRT-II preferentially localizes to late endosomal membranes, the ESCRT-II dependency of HBV and ASV may be related to their particular budding sites.

The results of our coimmunoprecipitation studies, demonstrating a physical interaction between core and ESCRT-II, substantiate the ESCRT-II dependency of HBV. Against expectation, however, ESCRT-II appears to assist in steps preceding the budding reaction of HBV, as evidenced by the potent decrease of pgRNA-containing capsids in ESCRT-II-depleted cells. While the deficiency of ESCRT-II does not impair the capsid assembly reaction, it selectively compromises the formation and/or stability of nucleocapsids. To account for the inhibitory effect of dysfunctional ESCRT-II, at least two possibilities may be considered. First, the molecular events accompanying nucleocapsid formation have been well characterized [28], [29], [31], but the temporal and spatial order of these events is less clear. Hence, ESCRT-II may be recruited to the budding site by nucleocapsid precursors, like e.g. core dimers, rather than by preassembled nucleocapsids, in order to assist during packaging of the pgRNA/P complex into the forming capsid. In the case of HIV-1, it has been reported that the nucleocapsid-retroviral RNA interaction is essential for structural particle stability [56]. Accordingly, the ESCRT-II assistance in HBV formation may not only affect nucleocapsid formation but also stable particle architecture. By virtue of its ability to bind the ESCRT-III subunit CHMP6 [8], [9], [22], ESCRT-II could next recruit ESCRT-III/VPS4 for HBV budding. In this scenario, ESCRT-II would perform functions that appear to be related to MVB sorting. On the other hand, however, it is equally possible that ESCRT-II may perform a “noncanonical” function during HBV maturation, like e.g. involving RNA guidance.

By analyzing the effects of deficit ESCRT-II on the sequential steps in the viral replication cycle, we find that it does not interfere with the transcription of the HBV genome and the translation of the viral structural proteins. Because the pgRNA not only serves as a replication intermediate but also as mRNA for the core and P protein, we infer from the unchanged core level that the synthesis and nuclear export of the pgRNA is not compromised in ESCRT-II depleted cells. Rather, subsequent steps including trafficking of the pgRNA within the cytoplasm and its selective encapsidation may be escorted by ESCRT-II. A role for ESCRT-II in mRNA transport has been documented in *Drosophila* in which the entire complex, via the GLUE domain of EAP45, binds to *bicoid* mRNA and establishes the mRNA gradient responsible for generating polarity in the embryo [57]. This activity does not require ESCRT-I and ESCRT-III, suggesting that the function of ESCRT-II in *bicoid* mRNA localization is unrelated to its role in endosomal protein sorting [57]. Studies in *Xenopus* revealed that ESCRT-II is a general mRNA binding protein and may function as a type of “RNA-Histone” to naked RNA, indicating that ESCRT-II may also have a role in mRNA localization in vertebrates [58|. The involvement of mammalian ESCRT-II in RNA transport events has been reported in HIV-1-replicating cells in which the absence of ESCRT-II led to a block to viral genomic RNA trafficking and hence to a decrease in virus assembly [50]. Based on these observations it is tempting to suppose that ESCRT-II may also direct the fate of the HBV pgRNA (or the pgRNA/P complex), such as influencing its stability and appropriate localization and/or providing its linkage to the viral core protein for encapsidation. Understanding how ESCRT-II mechanistically operates during HBV maturation will be an important next step forward.
